# Does the SARS-CoV-2 mRNA vaccine and its serum IgG levels affect fertility treatments and obstetric outcomes? An observational cohort study

**DOI:** 10.1007/s10238-024-01345-9

**Published:** 2024-04-23

**Authors:** Netanella Danielli Miller, Nitzan Goren Gepstein, Dovev Cohen, Einat Haikin Herzberger, Hila Shalev Ram, Jordana Mashiach Friedler, Maya Sharon Weiner, Roni Rahav, Victoria Indenbaum, Yaniv Lustig, Amir Wiser

**Affiliations:** 1 IVF Unit, Department of Obstetrics and Gynecology, Meir Medical Center, 4428164 Kfar Saba, Israel; 2 School of Medicine, Tel Aviv University, Tel Aviv, Israel; 3 Central Virology Laboratory, Public Health Services, Ministry of Health, Chaim Sheba Medical Center, Tel Aviv, Israel

**Keywords:** mRNA COVID-19 vaccine, Corona virus, In vitro fertilization, Semen

## Abstract

**Background:**

Although there are some data regarding the COVID-19 vaccine and in in vitro fertilization (IVF) treatments, its potential impact in terms of serum immunoglobulin G (IgG) levels has not been evaluated prospectively. This study aimed to assess the effect of COVID-19 vaccine and IgG levels on IVF outcomes.

**Methods:**

This observational, cohort study was conducted at a referral IVF unit. Couples undergoing IVF treatment during the COVID-19 vaccination period were recruited from March–April 2021. The study compared 38 women who had received the Pfizer mRNA COVID-19 vaccination to 10 women who had not and were not infected by the virus. We also compared pre- and post-vaccination IVF treatments for 24 women. The relation between serologic titers and IVF treatment outcomes was also assessed.

**Results:**

No significant difference was found between the vaccinated and unvaccinated/uninfected groups regarding the main outcome measures. However, there was a trend toward a higher pregnancy rate for the unvaccinated group (57% vs. 23%, *p* = 0.078) but no difference in delivery rate (*p* = 0.236), gestational week (*p* = 0.537) or birth rate (*p* = 0.671).

**Conclusion:**

We cautiously state that the COVID-19 mRNA vaccine does not affect fertility outcomes, including fertilization, pregnancy and delivery rates, obstetric outcomes, and semen parameters, regardless of measured IgG levels.

## Background

The coronavirus disease 2019 (COVID-19) is an ongoing global pandemic caused by the severe acute respiratory syndrome coronavirus (SARS-CoV-2) [1]. COVID-19 has a high prevalence [2], long incubation period [3] and efficient transmission [4].

Vaccines are the most promising and effective solution for preventing infectious diseases [5]. At the end of 2020, the FDA issued its first emergency use authorization for the Pfizer mRNA BioNtech COVID-19 vaccine [6, 7], with a reported 94%–95% efficacy in preventing COVID-19 [8]. Other vaccines, such as Moderna (mRNA vaccine) and Janssen followed.

Inactive, toxin-free vaccines are considered safe during pregnancy [9]. Although animal studies also did not show adverse effects on female reproduction or fetal/embryonal development, the data are still limited [10, 11]. Some data regarding the effect of the COVID-19 vaccine on fertility have shown that the vaccine itself may not affect fertility outcomes in terms of the number of oocytes retrieved, blastulation rate and pregnancy rate [12].

Another study evaluated 36 couples before and after the vaccine and found that the SARS-CoV-2 mRNA vaccine did not affect patient performance or ovarian reserve in their immediate subsequent IVF cycle [13]. However, it did not include serologic tests to evaluate the vaccination status of the couples. Moreover, few studies have tried to evaluate the effect of the COVID-19 vaccine and the IgG levels on treatment outcomes.

Given this, the current study examined the effect of COVID-19 vaccines on women and men undergoing IVF treatments. We investigated the following: a) differences in IVF treatment outcomes and semen analyses between vaccinated and unvaccinated/uninfected patients, b) differences in treatment outcomes before and after the vaccine among vaccinated patients, and c) correlations between IVF treatment outcomes and humoral response among vaccinated patients.

We believe that understanding the effects of the vaccine allows women and men to receive accurate advice and make informed decisions regarding COVID-19 vaccination and fertility treatments.

## Materials and methods

### Study design

This observational cohort study included women and men who were vaccine recipients and those who were neither vaccinated nor infected and were undergoing IVF treatments in a secondary medical center. We conducted serologic tests for all participants to exclude those who were not vaccinated and had been infected unknowingly.

### Study population

Couples undergoing IVF treatment during the COVID-19 vaccination period were recruited in March and April 2021. Eligibility criteria were ages 18–45 years and a well-documented COVID-19 vaccination for the vaccinated group. Exclusion criteria included women who did not plan embryo transfer (surrogacy, social or medical fertility preservation). Women with a positive SARS-CoV-2 polymerase chain reaction (PCR) test were also excluded.

Participants who met the eligibility criteria signed an informed consent and blood was drawn for serology. Women who were not vaccinated and had a positive serology test were also excluded. Demographic and fertility information were obtained from the electronic medical records.

### Serology assays

Samples from participants were tested with an enzyme-linked immunosorbent assay (ELISA) that detects IgG antibodies against the receptor-binding domain (RBD) of SARS-CoV-2. Titers > 1.1 were defined as positive.

A SARS-CoV-2 pseudo-virus neutralization assay was performed using a propagation–competent–spike [14], which was kindly provided by Gert Zimmer, University of Bern, Switzerland. Sera unable to reduce viral replication by 50% at a 1–8 dilution or below were considered non-neutralizing. All samples positive for RBD-IgG were tested for neutralization assay. Negative RBD-IgG tests were not tested since these have been shown to yield negative neutralization assay tests.

### Measures

Fertility-related outcomes of interest included total drug dose for induction, endometrial thickness, number of retrieved oocytes, estradiol and progesterone levels on the day of triggering, the ratio between estradiol on trigger day per retrieved oocyte, fertilization rate, embryo quality, number of blastocysts and clinical pregnancy rate. We also evaluated the semen total motile count (TMC), which is calculated by multiplying volume by concentration (million sperm/ml) by motility (percent moving). Clinical pregnancy was defined as one with a high concentration of human chorionic gonadotrophin and ultrasound confirmation of a gestational sac and was calculated as a percentage from women who had gone through embryo transfer.

The outcomes of vaccinated and unvaccinated/uninfected patients were compared. The latest cycle for the unvaccinated and the cycle at least one week post-vaccination were included. We confirmed that the treatment was at least one week after vaccination, as antibody levels are detectable at least 7 days after the second vaccination [15].

For vaccinated patients, treatment outcomes were compared between the most recent treatments before and after the vaccine. In addition, the associations between serologic IgG levels and neutralizing levels and fertility outcomes were evaluated. Also defined was a group of women with high IgG antibody levels (cut-off defined as the level above the median of IgG antibodies of the vaccinated group) who were compared to patients with low IGG levels.

### Statistical analysis

Data were analyzed using SPSS 24.0 for Windows (IBM Corp., Armonk, NY). Discrete variables are presented as numbers and percentages, and continuous variables as means and standard deviations (SD). We calculated *p* values using t test or chi-squared for continuous and categorical variables, respectively. Pearson’s coefficients were calculated between relevant variables. Multivariate regression was conducted to evaluate variables affecting the number of oocytes retrieved, fertilization rate and pregnancy rate. To compare pre- and post-vaccination, we used a paired t test. For all tests, a *p* value < 0.05 was considered significant.

## Results

**Fig. 1:**
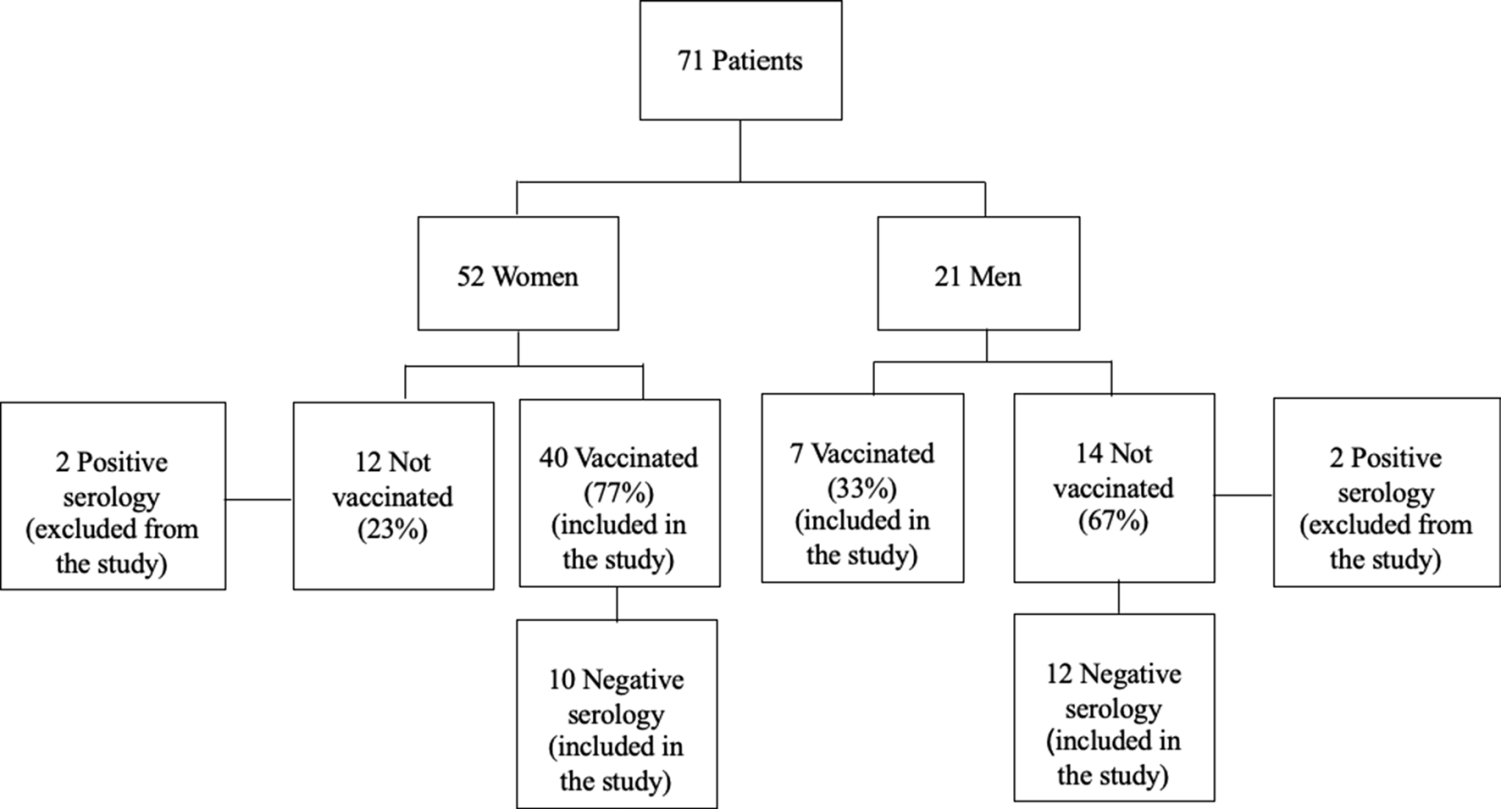
Flow diagram of cases included in the study

The cohort included 52 women and 21 men. Among the women, 40 were vaccinated (77%) and 12 were not (23%). Of the men, 7 were vaccinated (33%) and 14 were not (67%). After serology testing, 2 women (17%) and 2 men (14%) in the unvaccinated group were found to have positive serology and were excluded from the analysis. In the vaccinated group, 2 women whose treatment started less than a week after the second vaccine were also excluded. Figure 1 presents a flow diagram of cases included in the study.

### Comparison between vaccinated and unvaccinated/uninfected

The cohort included 38 vaccinated women and 10 who were not vaccinated or infected. Table 1 shows the basic characteristics and the fertility outcomes between groups. No differences in BMI (*p* = 0.108) or marital status were found (*p* = 0.198). There was a trend toward older age among the women in the vaccinated group compared to the non-vaccinated (38 ± 4.4 years vs. 35 ± 5.8 years, *p* = 0.083). No significant differences were found between groups regarding total drug dose for induction, endometrial thickness, number of retrieved oocytes, estradiol and progesterone levels on the triggering day, ratio between estradiol on triggering day per retrieved oocyte, fertilization rate, or embryo quality (Table 1). There was a trend toward higher pregnancy rate for the unvaccinated group (57% vs. 23%, *p* = 0.078). However, multivariable logistic regression for pregnancy rate showed no differences regarding fertilization rates (*p* = 0.842) or pregnancy rates (*p* = 0.414) between vaccinated and unvaccinated women. No difference was found between groups in terms of missed abortion rate (*p* = 0.125) and delivery rate (*p* = 0.236). Additionally, regarding obstetric outcomes, we did not find any significant differences in gestational week (*p* = 0.537) or birth weight percentile (*p* = 0.671; Table 2).

**Table 1:** Basic characteristics and fertility and obstetric outcomes of vaccinated and unvaccinated patients (women)

Characteristic	Vaccinated *N* = 38	Not vaccinated/not infected *N* = 10	*p* value
*Demographics*			
Female age, years (mean ± SD)	38 ± 4.4	35 ± 5.8	0.083
BMI (mean ± SD)	26.9 ± 5.7	23.4 ± 5.1	0.108
Marital status (% married)	71	89	0.198
Male age, years (mean ± SD)	41.2 ± 5.6	40 ± 4.6	0.714
Baseline FSH (IU)	8.8 ± 3	7.5 ± 3.7	0.363
*Fertility outcomes*			
Total dose of induction drug, pg/ml (mean ± SD)	2906 ± 1191	3290 ± 2828	0.717
Estradiol on triggering day, pg/ml (mean ± SD)	1619 ± 1077	1484 ± 997	0.721
Progesterone on triggering day, pg/ml (mean ± SD)	0.73 ± 0.72	0.39 ± 0.32	0.156
Endometrial thickness, mm (mean ± SD)	9.3 ± 2	9.1 ± 1.5	0.725
Number of oocytes (mean ± SD)	7.8 ± 5	7.7 ± 4.9	0.964
Estradiol/oocytes retrieved (mean ± SD)	267 ± 132	245 ± 108	0.645
Fertilization rate (%)	58	52	0.536
Embryo grade (mean ± SD)	3.1 ± 0.7	3 ± 1	0.62
Blastocyst (%)	29	43	0.491
ET occurred n (%)	30 (79%)	7 (70%)	0.347
Clinical Pregnancy n (%)	7 (23)	4(57)	**0.078**
Missed abortion rate n (%)	3 (42%)	0 (0%)	0.125
Delivery Rate n (%)	4 (57%)	4 (100%)	0.236
Gestational week (mean ± SD)	38.5 ± 1.2	39 ± 0.8	0.537
Birth Weight Percentile (mean ± SD)	41 ± 20	49.5 ± 27	0.671

**Table 2:** Basic characteristics and semen outcomes between vaccinated and non-vaccinated patients (men)

Characteristic/Outcomes	Vaccinated *N* = 7	Not vaccinated/not infected *N* = 12	*p* value
Male age, years (mean ± SD)	39.4 ± 7.5	35.6 ± 7.1	0.389
Concentration after centrifugation million/ml	65 ± 107	137 ± 165	0.287
Volume after centrifugation, ml	0.18 ± 0.19	0.19 ± 0.18	0.989
Total motile count	7.9 ± 10	5.4 ± 12	0.711

Semen analysis did not find any differences in the TMC between men who were vaccinated and those who were not (*p* = 0.711; Table 2).

### Comparison between pre- and post-vaccination treatment outcomes

Data regarding pre- and post-vaccine treatment were available for 24 of the 40 vaccinated patients. The mean interval between ovum pick-up cycles and the second vaccination was 33 days (range 11–69 days).

Data regarding the IVF outcomes before and after the SARS-CoV-2 mRNA vaccine are shown in Table 3. No significant differences were found regarding total drug dose for induction, endometrial thickness, number of retrieved oocytes, estradiol and progesterone on the day of triggering, the ratio between estradiol on trigger day per retrieved oocyte, fertilization rate, embryo quality, number of blastocysts and clinical pregnancy rate.

**Table 3:** Fertility outcomes pre- and post-vaccination

Fertility outcomes	Pre-vaccination *N* = 24	Post-vaccination *N* = 24	*p* value
Total dose of induction drug, pg/ml (mean ± SD)	3091 ± 1448	3156 ± 1233	0.802
Estradiol, pg/ml on triggering day (mean ± SD)	1689 ± 921	1582 ± 970	0.715
Progesterone, ng/ml on triggering day (mean ± SD)	0.5 ± 0.3	0.6 ± 0.6	0.274
Endometrial thickness, mm (mean ± SD)	9.8 ± 2.2	9.6 ± 2	0.53
Number of oocytes (mean ± SD)	7.5 ± 5	7.8 ± 4.8	0.805
Estradiol/oocytes retrieved (mean ± SD)	295 ± 218	264 ± 129	0.507
Fertilization rate (%)	60	52	0.364
Embryo grade (mean ± SD)	3.2 ± 0.6	3.1 ± 0.7	0.725
Blastocyst (%)	57	38	0.214
Clinical pregnancy (%)	22	17	0.747

### Correlation between serum IGG levels and fertility outcomes

All 40 vaccinated patients were found to have neutralizing antibodies (above 16), defined as a positive vaccination status [14].

The only positive correlation found was between neutralization titer and progesterone level on the day of induction (Pearson = 0.787, *p* = 0.001; Fig. 1). We also conducted a secondary analysis of women with high vs. low IGG levels. No difference was found regarding any of the measured fertility outcomes (data not shown).

## Discussion

Reluctance of women and men of fertility age to receive a new vaccine is common, especially due to uncertainty regarding its possible long-term effects and when a new vaccine, such as the mRNA COVID-19, is produced and approved emergently [16]. Yet, lack of knowledge or misleading information may cause uncertainty. Thus, it is essential to evaluate the safety of the COVID-19 mRNA vaccine regarding fertility and sterility.

The current study did not find any differences in fertility treatment or obstetric outcomes between women who were vaccinated and those who were not. In addition, no differences were found among patients before and after receiving the vaccine.

Our results support those of Bentov et al. who showed that neither COVID-19 infection, the BNT162b2 mRNA vaccine, nor the immune response, resulted in any measurable detrimental effects on IVF treatment and outcome parameters [17]. Moreover, Orvieto et al. showed that the mRNA SARS-CoV-2 vaccine did not affect patient performance or ovarian reserve in the immediate, subsequent IVF cycle [13]. Aharon et al. reported similar results [12]. However, it is important to mention that the authors did not conduct serology tests to exclude unvaccinated patients who might have been exposed to the virus.

A study of frozen embryo transfer cycles compared implantation rates among women who were SARS-CoV-2 vaccine seropositive, infection seropositive or seronegative. No difference was found in serum-documented hCG implantation rates or sustained implantation rates among the 3 groups [18]. We also evaluated pregnancy rates between vaccinated and unvaccinated/uninfected patients. Although we found a trend toward a higher pregnancy rate, this was not sustained in multivariant regression.

The lack of negative effects of the vaccine may be related to its biological activity, as it is composed of nucleoside-modified RNA (modRNA) [19] encoding the SARS-CoV-2 full-length spike, modified by two proline mutations. mRNA-based therapy avoids deleterious side effects (which include integration into chromosomes) that limit clinical application of most virus- and DNA-based vectors [20]. Other mRNA-based vaccines that have been investigated primarily with animals, including influenza A virus [21], rabies virus [22], HIV-1 [23], and Ebola virus [24], also showed efficacy of the mRNA vaccines combined with safety data.

The current study found no differences between semen analysis among vaccinated and unvaccinated men. Orvietto et al. also did not find any differences in semen volume, sperm concentration, sperm %, and pre-wash TMC, based on vaccine status [13]. This may be explained by the biological activity of the vaccine, as mentioned above [20]. In addition, spermatogenesis takes 74 days and another 12–21 days to be transported through the epididymis to the ejaculatory ducts [25]. Thus, the specific semen analysis examined may have represented sperm parameters before exposure to the vaccine.

This study supports the approach of major professional associations. The most recent SRM, ACOG and SMFM Joint Statement notes that medical experts continue to assert that COVID-19 vaccines do not affect fertility [26].

When evaluating IgG serology titers, the only effect on fertility outcomes that we found was a positive relation between progesterone levels on the day of triggering and IgG titer. Bentov et al. also found that serum progesterone was lower in the non-exposed group compared to the exposed group [17]. Progesterone is known to be involved in the immune response. Progesterone receptors are expressed in most immune cells, including epithelial cells, macrophages, dendrites, lymphocytes, mast cells, and eosinophils, and help modulate the immune response to pathogens [27]. Moreover, women are known to have higher levels of estrogen and progesterone, which have been shown to modulate a more robust immune response [27]. During the COVID-19 pandemic, this study suggested giving hormone replacement therapy, including estrogen and progesterone, to older patients based on the evidence that sex hormone levels can influence immune system function [28]. Accordingly, it may be suggested that the higher immune response in some women may have triggered activation of progesterone as an immune system modulator. Further studies are needed to evaluate this issue.

The strengths of this study relate to the prospective evaluation of the important question regarding whether mRNA COVID-19 vaccination affects fertility treatments. Evaluation of the serologic titer was also very important. Moreover, we evaluated treatment measures, pregnancy and obstetric outcomes and semen analyses. It was also important that we evaluated the serologic status of each patient to exclude exposed, unvaccinated patients; rendering our analyses more accurate and precise. Therefore, to avoid selection bias, it was essential to exclude these individuals when analyzing the unvaccinated population. However, this study was limited by its relatively small sample size. In addition, it is essential to evaluate long-term pregnancy outcomes, congenital malformations. Future, larger studies will be needed to validate our observations and to maintain longer follow-up of these patients.

## Conclusions

We cautiously state that the COVID-19 mRNA vaccine does not affect fertility outcomes, including fertilization, pregnancy and delivery rates, obstetric outcomes, and semen parameters, regardless of the IgG levels. Moreover, no relation to IgG titers and fertility outcomes was found, except for higher progesterone levels on triggering day. Larger, prospective studies are needed to validate these observations.

## Data Availability

The data presented in this study are available upon request from the corresponding author. The data are not publicly available due to privacy limitations.
